# The modified NCEP ATP III criteria maybe better than the IDF criteria in diagnosing Metabolic Syndrome among Malays in Kuala Lumpur

**DOI:** 10.1186/1471-2458-10-678

**Published:** 2010-11-06

**Authors:** Foong Ming Moy, Awang Bulgiba

**Affiliations:** 1 Julius Centre University of Malaya, Department of Social & Preventive Medicine, Faculty of Medicine, University of Malaya, 50603 Kuala Lumpur, Malaysia

## Abstract

**Background:**

Metabolic Syndrome is associated with increased risk for type 2 diabetes and cardiovascular diseases. However, different diagnostic criteria have been recommended by different expert groups. In Malaysia, there is a lack of research comparing these different diagnostic criteria. Therefore, it is our aim to study the concordance between the IDF and the modified NCEP ATP III definitions of Metabolic Syndrome among a Malay cohort in Kuala Lumpur; and to demonstrate if all participants have the same cardiometabolic risks.

**Methods:**

This was an analytical cross sectional study. Ethics approval was obtained and informed consent was given by all participants. Anthropometric measurements, blood pressure, fasting blood glucose and lipid profile were taken following standard protocols.

**Results:**

Metabolic Syndrome was diagnosed in 41.4% and 38.2% participants using the modified NCEP and IDF criteria respectively. Among those diagnosed with Metabolic Syndrome by modified NCEP, 7.6% were missed by the IDF criteria. Participants diagnosed by the modified NCEP criteria had lower BMI and waist circumference but had higher cardiometabolic risks than those diagnosed with both criteria. Their blood pressure, glucose, total cholesterol and triglyceride were more adverse than the IDF group. This demonstrated that central obesity may not be a prerequisite for the development of increased cardiometabolic risks within this Malay cohort.

**Conclusion:**

Metabolic syndrome is common in this Malay cohort regardless of the criterion used. The modified NCEP ATP III criteria may be more suitable in diagnosis of metabolic syndrome for this Malay cohort.

## Background

Metabolic Syndrome has been demonstrated as a common precursor to the development of type 2 diabetes and cardiovascular disease (CVD)[1] as well as a risk factor for all cause mortality[2]. Individuals with Metabolic Syndrome are associated with approximately five and two-fold increased risk for type 2 diabetes and CVD respectively[1]. Metabolic syndrome has also been linked with obesity and a sedentary lifestyle, both of which are modifiable [3]. More effort should be given to promoting a healthy lifestyle with increased physical activity and reduced obesity [1,4] . Individuals with metabolic syndrome should be identified early so that their cardiovascular risk factors can be reduced[5].

Although there is general consensus that obesity and metabolic syndrome requires greater attention, there is disagreement over the diagnostic criteria of metabolic syndrome. Different criteria used for diagnosing Metabolic Syndrome provide differing results. Expert groups from the International Diabetes Federation (IDF), National Cholesterol Education Program Adult Treatment Panel III (NCEP ATP III), World Health Organisation (WHO) etc have different diagnostic criteria [1,6,7].

In Malaysia there is a paucity of studies comparing the different diagnostic criteria with the modified waist circumference cut-off values which are ethnic and gender specific as recommended by IDF and the modified NCEP ATP III criteria. We therefore set out to study the concordance between these two definitions of Metabolic syndrome among a Malay cohort in Kuala Lumpur; and to demonstrate if participants identified by the modified NCEP ATP III criteria but not by the IDF criteria have the same cardio-metabolic risks.

## Methods

### Study population

This was an analytical cross-sectional study. The study population was the Malay employees from a health screening program of a public university in Kuala Lumpur. All eligible Malay employees were invited to take part in the study. A total of 1494 Malay employees participated in the study, giving a response rate of 85%. All participants were aged 35 years and above as this screening program only included staff of this age range. Approval was obtained (reference number MEC 782.18) from the Medical Ethics Committee of the medical centre. This committee is responsible for ethical issues in all research projects involving humans conducted by Medical Faculty staff of the university. Approval was also obtained from the management of the university. Written informed consent was given by all participants.

### Data Collection

Data was collected over two years (2008-2009) in the university campus. Measurements included anthropometric measurements (weight, height, waist and hip circumference); systolic and diastolic blood pressure, fasting blood glucose and fasting lipid profile were also taken. Weight and height were measured using calibrated digital weighing scales and stadiometers respectively. The waist and hip circumferences were measured with circumference measurement tape. The waist was defined as the point midway between the iliac crest and the costal margin (lower rib); while the hip circumference was defined as being the widest circumference over the buttocks and below the iliac crest [8,9]. All measurements were conducted by trained staff and quality checks were conducted regularly.

Blood pressure was measured using a digital automatic blood pressure monitor (Omron HEM - 907 model) while lipid profile was analyzed using the Dimension^® ^clinical chemistry system which was an in-vitro diagnostic test. All biochemical analysis was conducted by the Clinical Diagnostic Laboratory of the medical centre from the same university. Body Mass Index (BMI) was derived following the formula of weight in kg/height^2 ^in meters. We used a self-reported diagnosis of diabetes and hypertension in the study.

### Definition of Metabolic Syndrome

Metabolic syndrome was defined following the criteria provided by the modified NCEP ATP III and IDF groups. According to the modified NCEP criteria [1], the presence of any three of the following five factors is required for a diagnosis of Metabolic Syndrome: abdominal obesity, hypertriglyceridaemia (triglycerides ≥1.7 mmol/L); low HDL cholesterol (HDL cholesterol ≤1.03 mmol/L for men and ≤1.29 mmol/L for women); elevated blood pressure (systolic blood pressure ≥130 mmHg and/or diastolic blood pressure ≥85 mmHg or current use of antihypertensive drugs); impaired fasting glucose (fasting plasma glucose ≥5.6 mmol/L). The modified NCEP ATP III criteria suggested the cut-off points of waist circumference should be ethnic specific where individuals of Asian origin should use the cut-off of 90 cm in men and 80 cm in women. For NCEP criteria, abdominal obesity is a component of the syndrome but not a prerequisite for its diagnosis. The IDF's diagnosis of Metabolic Syndrome places emphasis on abdominal obesity as a required factor [10] plus any two of the other four criteria which are essentially identical to those provided by NCEP ATP III. The IDF criterion uses ethnic-specific waist circumference cut-off points as a requirement for diagnosis. Similar to the modified NCEP ATP III criteria, IDF recommends cut-off levels of 90 cm in men and 80 cm in women for central obesity among Asians. For both criteria, we used the recommended cut-off for Asians (90 cm in men and 80 cm in women) as there are no national cut-off values specific for Malaysia.

### Data Analysis

Data was entered and analysed using SPSS for Windows version 16.0. Categorical variables were presented as frequency and percentages while quantitative variables were presented as mean with 95% confidence interval where appropriate. Kappa statistics was used to measure agreement between the two criteria. Independent t test was used to compare the cardiometabolic risks among gender.

## Results

A total of 1494 Malay employees participated in the study with 697 (46.7%) males. Half of them were in their fifties (Table 1). About 5.5% of them were diagnosed with type II diabetes mellitus while 12.2% were hypertensive.

**Table 1:** Prevalence of Metabolic Syndrome and baseline characteristics by IDF or modified NCEP criteria

	Total population (n = 1494)	IDF (n = 571)	Modified NCEP (n = 618)
Prevalence (%)		38.2	41.4
Gender: Male	697 (46.7)	302 (52.9)	338 (54.7)
Female	797 (53.3)	269 (47.1)	280 (45.3)
Age group: Less than 40 years	191 (12.8)	61 (10.7)	61(9.9)
40 - 49 years	774 (51.8)	270 (47.3)	300 (48.5)
50 - 59 years	493 (33.0)	227 (39.8)	243 (39.3)
60 years and above	36 (2.4)	13 (2.3)	14 (2.3)
Diabetes mellitus	82(5.5)	61 (10.7)	69 (11.2)
Hypertension	183 (12.2)	103 (18.0)	109 (17.6)

Metabolic Syndrome was diagnosed in 618 (41.4%) and 571 (38.2%) participants using the modified NCEP and IDF criteria respectively (Table 1). The prevalence of Metabolic Syndrome among males and females were 54.7% and 45.3% for the modified NCEP criteria; and 52.9% and 47.1% for IDF criteria respectively. There were 47 (36 males and 11 females) or 7.6% of all participants who were diagnosed by the modified NCEP criteria but missed by the IDF criteria. Those participants missed by the IDF criteria were mainly males (76.6%). There were no participants who were diagnosed by IDF but missed by the modified NCEP criteria. Among those diagnosed to have Metabolic Syndrome, 92% of participants were identified equally by both criteria. The prevalence of Metabolic Syndrome was highest among those aged 40-49 years, followed by those aged 50-59 years. The prevalence among those aged 60 years and older was the lowest among all age groups (Table 1). About 11% of the participants diagnosed with Metabolic Syndrome were diabetics while 18% were hypertensive. There was no significant difference in the prevalence of diabetes or hypertension observed in those diagnosed as having Metabolic Syndrome by either IDF or modified NCEP criteria. The agreement of these two criteria as shown by the Kappa statistics was 0.93.

Table 2 compares cardio-metabolic risk factors of Metabolic Syndrome diagnosed by the IDF and modified NCEP criteria. Participants who were not diagnosed by both criteria were significantly younger than the other two groups (either diagnosed by the modified NCEP only or diagnosed by both the modified NCEP and IDF criteria). They also had the lowest levels of systolic and diastolic blood pressure, glucose and lipid profiles. BMI and waist circumferences reflecting overall obesity and central obesity were significantly lower among those diagnosed by the modified NCEP criteria only. Systolic and diastolic blood pressure, total cholesterol, LDL-cholesterol, glucose and triglyceride were found to be highest while HDL-cholesterol was lowest among the modified NCEP group.

**Table 2:** Comparison of cardiometabolic risk factors of metabolic syndrome by modified NCEP and IDF criteria

	NCEP only Mean (95% CI) (n = 47)	Both (NCEP and IDF) Mean (95% CI) (n = 571)	Neither (NCEP or IDF) Mean (95% CI) (n = 876)
Age (years)	49.1 (47.7, 50.4)	48.3 (47.8, 48.9)	47.0 (46.6, 47.4)
BMI	24.2 (23.6, 24.9)	29.5 (29.2, 29.9)	25.7 (25.4, 25.9)
Waist (cm)	82.8 (81.3, 84.3)	95.6 (94.8, 96.3)	84.6 (83.9, 85.3)
Systolic blood pressure (mmHg)	142.2 (137.3, 146.9)	139.4 (137.9, 140.9)	125.4 (124.3, 126.6)
Diastolic blood pressure (mmHg)	87.9 (85.1, 90.8)	86.7 (85.8, 87.6)	78.1 (77.4, 78.8)
Glucose (mmol/l)	8.4 (7.0, 9.8)	6.6 (6.4, 6.9)	4.9 (4.9, 5.2)
Total cholesterol (mmol/l)	5.9 (5.6, 6.3)	5.6 (5.6, 5.7)	5.5 (5.5, 5.6)
Triglyceride (mmol/l)	2.7 (2.3, 3.1)	2.1 (1.9, 2.2)	1.1 (1.1, 1.2)
HDL-cholesterol (mmol/l)	1.0 (0.9, 1.1)	1.1 (1.0, 1.1)	1.4(1.3, 1.4)
LDL- cholesterol (mmol/l)	3.7 (3.4, 4.0)	3.6 (3.5, 3.7)	3.6 (3.5, 3.7)
			

CI: Confidence Interval

BMI: Body Mass Index, HDL: High Density Lipoprotein, LDL:Low Density Lipoprotein

Stratified analysis of the above cardio-metabolic risk factors by sex was also conducted to explore if there was any gender difference observed in all sub-groups (Table 3). In the modified NCEP group, females were found to have more adverse levels of fasting blood glucose, systolic and diastolic blood pressure; while the males were found to have higher levels of total cholesterol, triglyceride and LDL-cholesterol. Mean BMI was also higher in females. However, all these observed differences were not statistically significant (p > 0.05). The only significant gender differences observed were waist circumference (higher in males) and HDL-cholesterol level (higher in females). In the group diagnosed by both modified NCEP and IDF criteria, females were significantly (p < 0.01) more obese and had higher HDL-cholesterol while males had larger waist circumference (p < 0.001) and higher triglyceride level (p < 0.001). Although there were no significant gender difference observed in other cardiometabolic risk factors, systolic blood pressure and total cholesterol levels were higher among males in this group. On the other hand, males who were not diagnosed with either criterion had significantly higher mean levels of cardiometabolic risk factors than their counterparts.

**Table 3:** Comparison of cardiometabolic risk factors by sex using modified NCEP and IDF criteria

		NCEP only (n = 47) (Male = 36, Female = 11)		Both (NCEP and IDF) (n = 571) (Male = 295, Female = 266)		Neither (NCEP or IDF) (n = 876) (Male = 517, female = 359)	
		Mean ± s.d.	p	Mean ± s.d.	p	Mean ± s.d.	p
Age (years)	Male	49.1 ± 4.4	0.98	48.7 ± 6.6	0.17	47.6 ± 6.7	0.02
	Female	49.1 ± 5.5		48.0 ± 5.6		46.6 ± 5.9	
							
BMI	Male	24.0 ± 2.3	0.20	28.5 ± 3.4	<0.001	25.4 ± 4.1	0.03
	Female	25.0 ± 2.0		30.8 ± 5.0		25.9 ± 4.4	
							
Waist (cm)	Male	84.9 ± 3.5	<0.001	97.6 ± 7.9	<0.001	88.5 ± 10.9	<0.001
	Female	75.9 ± 2.6		93.3 ± 9.8		81.9 ± 10.1	
							
Systolic blood pressure (mmHg)	Male	140.4 ± 14.6	0.19	139.9 ± 17.0	0.46	129.3 ± 16.7	<0.001
	Female	147.9 ± 21.3		138.8 ± 19.5		122.8 ± 16.3	
							
Diastolic blood pressure (mmHg)	Male	87.0 ± 8.4	0.35	86.6 ± 10.6	0.85	79.8 ± 10.4	<0.001
	Female	91.0 ± 12.9		86.8 ± 11.3		76.9 ± 10.6	
							
Glucose (mmol/l)	Male	8.1 ± 4.9	0.34	6.6 ± 2.8	0.96	5.1 ± 1.0	0.01
	Female	9.6 ± 4.6		6.6 ± 2.9		4.9 ± 0.8	
							
Total cholesterol (mmol/l)	Male	6.1 ± 1.2	0.20	5.7 ± 1.1	0.15	5.6 ± 0.9	0.02
	Female	5.6 ± 0.8		5.6 ± 1.1		5.5 ± 1.2	
							
Triglyceride (mmol/l)	Male	2.9 ± 1.4	0.07	2.3 ± 1.1	<0.001	1.4 ± 0.7	<0.001
	Female	2.1 ± 0.8		1.8 ± 1.2		0.9 ± 0.4	
							
HDL-cholesterol (mmol/l)	Male	1.0 ± 0.1	<0.001	1.1 ± 0.2	<0.001	1.3 ± 0.2	<0.001
	Female	1.2 ± 0.2		1.2 ± 0.2		1.5 ± 0.3	
							
LDL- cholesterol (mmol/l)	Male	3.9 ± 1.1	0.12	3.6 ± 1.0	0.51	3.8 ± 0.9	<0.001
	Female	3.2 ± 1.1		3.6 ± 0.9		3.5 ± 0. 8	

CI: Confidence Interval; s.d: standard deviation

BMI: Body Mass Index, HDL: High Density Lipoprotein, LDL:Low Density Lipoprotein

## Discussion

The overall prevalence of Metabolic Syndrome in this cohort was high regardless of any criteria used. This cohort's older age and its Malay ethnicity may partly explain this high prevalence. Preliminary results derived from a rural area with a predominantly Malay population in Malaysia suggested that metabolic syndrome, as defined by the IDF criteria, affected an estimated 36.5% and 50.5% of adult males and females respectively [11], findings which were quite similar to ours. However, our results show a higher prevalence than Malays from Singapore within the same age groups [12].

We observe that the prevalence of Metabolic Syndrome increased with age but was reduced in the oldest age group. This contradicts results shown elsewhere [12,13]. The unexpected low prevalence among the oldest age group can either be attributed to the "healthy worker effect" or merely be a chance finding. More males were diagnosed with Metabolic Syndrome compared to females using either criterion as reported in a study among Singapore Malays [12]. However, a survey of rural Malays in Malaysia [11] demonstrated a higher prevalence of Metabolic Syndrome among females (50.5%) compared to males (36.5%). Working status may be the reason for this difference. All our female participants were gainfully employed while most females in the rural areas were housewives. In the latest Malaysian National Health & Morbidity Survey III (NHMS III) in 2006, a greater proportion of housewives were found to be obese compared to other occupation categories [14]. A lack of gainful employment, which may be associated with lower education and lower self-esteem, may in part explain this difference between our sample and that of rural Malay women.

Our results showed both the modified NCEP ATP III and IDF criteria similarly diagnosed 92.4% of participants as having Metabolic Syndrome. The Kappa statistics also suggested high agreement between these two criteria after correction for agreement by chance. Despite the above similarities and agreement in the diagnosis of Metabolic Syndrome, these two criteria provided different prevalence estimates and identify different individuals.

Similar to our findings, Lee et al and Xavier et al found higher prevalence of Metabolic Syndrome among Singaporeans and Japanese respectively using modified NCEP criteria compared to IDF criteria [13,15]. In another study among the Koreans, the IDF criteria too failed to identify 44.9% of men and 16.6% of women as having Metabolic Syndrome according to the modified NCEP criteria [16]. Those missed by the IDF criteria were predominantly males. This group of participants (identified by NCEP criteria alone) had lower BMI and waist circumference but at a higher cardio-metabolic risk than those diagnosed with both criteria. Their blood pressure, glucose, total cholesterol and triglyceride levels were more adverse than the other two groups. Similar results were found in other studies among Asians [16,17]. Due to the small sample size in the modified NCEP group, there was inadequate evidence to show if there was any gender difference in most of the cardiometabolic risk factors. The only observed difference was higher HDL-cholesterol levels among females and larger waist circumference among males which most probably may be due to gender specific physiological difference. On the other hand, males who were not diagnosed with either criterion had significantly higher mean levels of cardiometabolic risk factors than females. However, most of these risk factors were within normal ranges except for total cholesterol and LDL-cholesterol. This group of participants should be targeted for health education and promotion programs in the prevention of Metabolic Syndrome.

The proportions of participants with diabetes and hypertension were not significantly different in the modified NCEP group or group diagnosed by both criteria. This demonstrated that the adverse levels as observed in the modified NCEP group were not due to higher proportions of participants with diabetes and hypertension. We are of the opinion that central obesity may not be the prerequisite for the development of increased cardio-metabolic risks as reported elsewhere [13]. According to Lee at al [13], the definition of Metabolic Syndrome should have central obesity as an "optional" rather than "essential" criterion as this would identify more high risk individuals among the Asians. Similar recommendations were given in the recent Joint Scientific Statement in "Harmonizing the Metabolic Syndrome" [18] by the various expert groups. Their consensus was that there should not be an obligatory component. Any three abnormal findings out of five should suffice to diagnose a person as having Metabolic Syndrome. A single cut-off point would be used for all components except waist circumference where the interim national or regional cut-off points can be used. Within this Malay cohort, adverse cardio-metabolic risks were observed in those with lower BMI or waist circumference. Zahel et al [19] recommended waist circumference cut off value of 83 cm for both males and females to define overweight or obesity among adults in Malaysia. This recommended cut off value for males is lower than 90 cm while for females is higher than 80 cm as recommended by NCEP ATP III [1] and IDF [10]. We think further studies are required to determine the suitability of 90 cm and 80 cm as the optimal cut- off levels for central obesity in Malays.

As this is a cross-sectional study, we cannot examine which criterion for diagnosing Metabolic Syndrome has better predictive power in diagnosing diabetes, CVD and premature death. Follow-up studies are needed to examine the significance of Metabolic Syndrome following all criteria for the assessment of risk for diabetes and/or CVD. As this study population is from a public university, the findings may not be easily generalised to the whole Malay population of the country. However, it cannot be denied that this study is one of the few that compared the two different definitions of Metabolic Syndrome among a reasonably large sample in Malaysia. This will provide a basis for future and larger scale studies on this topic in Malaysia.

## Conclusion

In conclusion, Metabolic Syndrome is common in this Malay cohort in Kuala Lumpur regardless of the criteria used. The modified NCEP ATP III criteria may be more suitable in diagnosing Metabolic Syndrome in this Malay cohort. An effective intervention program should also be planned for this cohort as the complications of Metabolic Syndrome including diabetes and CVD, will become epidemic in the near future.

## Competing interests

The authors declare that they have no competing interests.

## Authors' contributions

FMM contributed to conceptualizing the paper, data entry, data analysis and writing of the manuscript while AB contributed in data analysis and writing of the manuscript. Both authors approved the final draft.

## Pre-publication history

The pre-publication history for this paper can be accessed here:

http://www.biomedcentral.com/1471-2458/10/678/prepub
